# In Fungal Intracellular Pathogenesis, Form Determines Fate

**DOI:** 10.1128/mBio.02092-18

**Published:** 2018-10-23

**Authors:** Robin C. May, Arturo Casadevall

**Affiliations:** a Institute of Microbiology & Infection, University of Birmingham, Birmingham, United Kingdom; b School of Biosciences, University of Birmingham, Birmingham, United Kingdom; c Department of Molecular Microbiology and Immunology, Johns Hopkins School of Public Health, Baltimore, Maryland, USA

**Keywords:** *Candida*, *Cryptococcus*, fungus, macrophage, phagosome

## Abstract

For pathogenic microbes to survive ingestion by macrophages, they must subvert powerful microbicidal mechanisms within the phagolysosome. After ingestion, *Candida albicans* undergoes a morphological transition producing hyphae, while the surrounding phagosome exhibits a loss of phagosomal acidity. However, how these two events are related has remained enigmatic. Now Westman et al. (mBio 9:e01226-18, 2018, https://doi.org/10.1128/mBio.01226-18) report that phagosomal neutralization results from disruption of phagosomal membrane integrity by the enlarging hyphae, directly implicating the morphological transition in physical damage that promotes intracellular survival. The *C. albicans* intracellular strategy shows parallels with another fungal pathogen, *Cryptococcus neoformans*, where a morphological changed involving capsular enlargement intracellularly is associated with loss of membrane integrity and death of the host cell. These similarities among distantly related pathogenic fungi suggest that morphological transitions that are common in fungi directly affect the outcome of the fungal cell-macrophage interaction. For this class of organisms, form determines fate in the intracellular environment.

## Abstract (precis)

For pathogenic microbes to survive ingestion by macrophages, they must subvert powerful microbicidal mechanisms within the phagolysosome. After ingestion, *Candida albicans* undergoes a morphological transition producing hyphae, while the surrounding phagosome exhibits a loss of phagosomal acidity.

## COMMENTARY

Candida albicans is the leading fungal killer of humans, with around 700,000 cases of systemic infection worldwide each year (1). Typically present as a commensal yeast within the healthy human microbiota, a key virulence trait of C. albicans is its ability to survive within and escape from host phagocytes.

It has been known for over 40 years (2) that, upon engulfment by host macrophages, C. albicans switches morphotype and begins to filament, eventually lysing the host cell and penetrating surrounding tissues. This is no mean feat, given that the macrophage phagosome is an extremely inhospitable place. In particular, within minutes of engulfment, the phagosomal pH begins to drop rapidly, limiting microbial growth and activating acid proteases within the compartment to degrade phagosomal contents.

To avoid this fate, several intracellular pathogens have evolved strategies to neutralize phagosomal Ph, and indeed this is true of C. albicans. Although the pH of the *Candida*-containing phagosome initially drops, it rapidly reneutralizes, an event that correlates with fungal filamentation and host cell lysis.

Like many biological processes, there is a molecular devil in the details of this process regarding the temporal relationship between phagosomal neutralization and escape. Specifically, the questions of whether phagosomal neutralization precedes fungal escape and whether this escape relies on physical rupture have remained unclear. Now Westman and colleagues (3) have provided a major development in this debate; by using advanced imaging approaches, they show that phagosomal neutralization relies on the fungus breaking the phagosomal membrane, most likely by physical pressure of filamentation, rather than through a biochemical neutralization process.

Previous data have demonstrated that C. albicans uses exogenous amino acids to generate ammonia (4–6), which can raise the pH of the surrounding media by 3 logs within a few hours. Mutants that are blocked in amino acid uptake fail to neutralize acidic environments and also fail to escape from macrophages (7), leading to the logical conclusion that biochemical neutralization of the phagosome by ammonia is required for phagosomal rupture.

This is logical, but now it turns out that this is perhaps not the full story. By combining old-fashioned biochemistry with 21st-century imaging, Westman et al. mapped out the movement of ions across the *Candida* phagosome membrane in unprecedented detail. What they revealed is that the ammonia generated by *Candida* leaks out rapidly of the phagosome through the membrane. At the same time, the macrophage V-type ATPase pumps protons into the phagosome at approximately three times the rate at which ammonia leaves. Consequently, straightforward acid/base neutralization cannot explain the raised pH of the C. albicans phagosome. Instead, the authors show that the fungal phagosome initially acidifies normally but then rapidly neutralizes a couple of hours later. By using so-called “yeast-locked” mutants, which cannot filament, they show that this neutralization relies on fungal filamentation. In other words, the fungus first forms a hypha, which ruptures the phagosomal membrane, allowing pH equilibration with the cytosol. Since filamentation is far more rapid at neutral pH, this rupture triggers rapid hyphal growth, eventually lysing the host cell and driving pathogenesis.

Like all good science, these new findings solve some mysteries but generate new ones. C. albicans was recently shown to produce a unique toxin, Candidalysin (8), which is essential for permeabilization and invasion of epithelial cells. However, Westman and colleagues show that Candidalysin plays very little role in phagosomal permeabilization, raising the intriguing possibility that C. albicans has evolved distinct approaches for rupturing different host membranes.

These new findings further complicate the already-complex interplay between *Candida* pathogenesis and host inflammatory signaling. The major route of escape for *Candida* from macrophages is by triggering host cell pyroptosis (9, 10). However, although fungal filamentation is necessary for this process, it is not sufficient (11, 12), and indeed recent data indicate that pyroptosis is not triggered by phagosomal rupture but rather by fungal cell wall remodelling within the phagosome (13, 14). This raises the intriguing question of why, if pyroptosis and escape can be triggered from within the phagosome, is phagosome rupture required at all?

How then, can these apparently conflicting sets of data be reconciled? A tempting model is that of metabolically driven changes to the *Candida* cell wall. Several environmental signals, including arginine, CO_2_, and urea, are required to trigger hyphal development in *Candida* (7). The availability of these molecules within the phagosome is strongly influenced by amino acid permeases and by free ion availability. For instance, arginine is converted by arginases (produced by both the fungus and the host cell) into urea, which is further broken down into ammonia and CO_2_. Although ammonia diffuses rapidly across the phagosome membrane, the localized production of CO_2_ may suffice to initiate filamentation. This morphological change will, in turn, necessitate fungal cell wall remodelling that ultimately triggers pyroptosis and host cell lysis. Consequently, filamentation, phagosomal rupture, and pyroptosis run in close parallel, such that phagosomal rupture sometimes precedes host lysis and sometimes does not.

### Broader understanding of fungal-macrophage interactions.

This new paper also raises some interesting parallels with another major human fungal pathogen: Cryptococcus neoformans. Although separated from Candida albicans by ∼400 million years of evolution (15), the two organisms share many remarkable features, including an ability to survive, grow, and ultimately escape from within the mammalian phagosome. For example, both organisms are able to exit the host phagocyte either via host cell lysis or via the enigmatic process known as vomocytosis or nonlytic exocytosis (16, 17). Both organisms are also able to rupture the phagosomal membrane, both undergo pH-dependent remodelling of the cell wall while intracellular, and both show activation of the inflammasome/pyroptosis (9, 18, 19).

**FIG 1:**
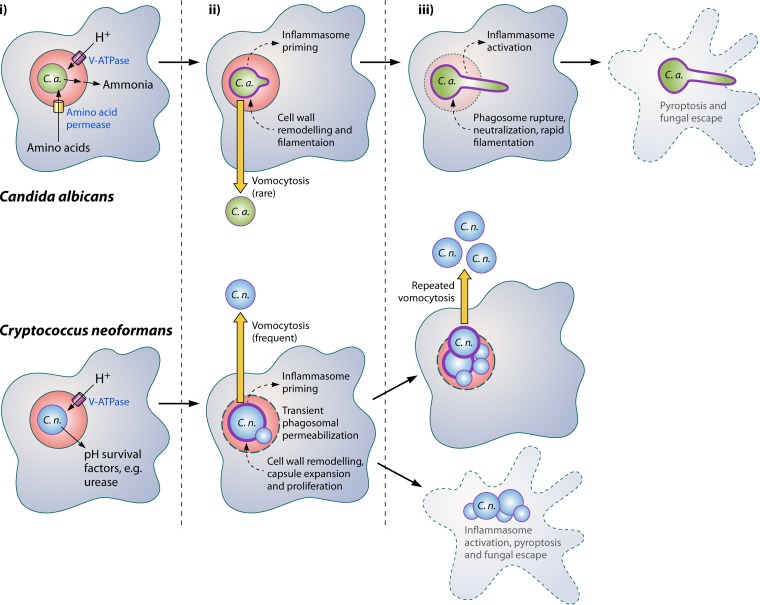
Intraphagosomal strategies in Candida albicans (top) and Cryptococcus neoformans (bottom). (i) Following uptake, the activity of the V-ATPase on the phagosome drives rapid acidification. C. neoformans expresses factors such as urease to help deal with this low pH, while C. albicans expresses amino acid permeases within the phagosome and uses imported amino acids to generate ammonia, although this diffuses rapidly across the phagosome membrane, leaving the phagosomal pH low. (ii) *Candida* then begins to filament, while *Cryptococcus* undergoes radial growth and capsule thickening. Both events are accompanied by cell wall remodelling, which activates the host inflammasome, and in both cases, the increase in fungal size exerts physical pressure on the phagosome membrane. (iii) In the case of cryptococci, repeated rounds of vomocytosis can reduce this intracellular burden, although this is rare for *Candida*. For both organisms, the eventual rupture of the phagosome membrane triggers full activation of the inflammasome and host cell pyroptosis, releasing the fungi back into the environment.

It is therefore appealing to propose a “unified model” for both fungal species, and perhaps others, in which the observed outcome of the macrophage-fungus interaction depends on the relative contributions of these different facets (Fig. 1). Following uptake, both organisms respond to the new (phagosomal) environment by remodelling their cell wall (and, in the case of *Cryptococcus*, their capsule). This remodelling alerts the host cell by activating (in a manner yet to be elucidated) the inflammasome and triggering responses that may ultimately include either vomocytosis/extrusion or pyroptosis/lysis. Here, the outcomes diverge based on the intracellular lifestyle of each fungus. In the case of *Cryptococcus*, this involves transient permeabilization of the phagosomal membrane, while for *C. albicans* it results in morphological change and filamentation. In rare cases, the host cell can expel *Candida* nonlytically early on (20), but in most instances, filamentation and phagosomal rupture occur before this event. Once ruptured, the phagosome instantaneously neutralizes, stimulating rapid extension of the C. albicans hyphae. The host cell is now irretrievably condemned; the sudden abundance of pathogen-associated molecular patterns (PAMPs) triggers pyroptosis and death, releasing the fungus.

For *Cryptococcus*, the lack of filamentation means that phagosomal persistence can go on for hours if not days with repeated cycles of phagosomal permeabilization, provided that leakage of its contents is not sufficient to irreversibly damage the host cell. This period most likely represents a stalemate between the two sides: if the host cell detects the fungus, it triggers either nonlytic expulsion or full inflammasome activation and fungal cell death. If the *Cryptococcus* can remain below the radar, then it can replicate, releasing multiple daughter cells into the environment once the host cell eventually lyses. However, if the phagosomal membrane is compromised, leakage of its contents into the cytoplasm can set forth a series of events that lead to host cell death (21, 22). In contrast to that of C. albicans, the cryptococcal phagosome does not immediately neutralize, an effect that might be due to the acid base properties of the cryptococcal capsule, which contains glucuronic acids (23). As with C. albicans, the compromise of the phagosomal membrane might involve a morphological change in the fungus, but unlike with the *Candida* shape change, for *Cryptococcus* spp. it involves capsular growth with radial enlargement. Intraphagosomal enlargement of the cryptococcal capsule would place tremendous stress on the integrity of the vesicular membrane, and C. neoformans phospholipases contribute to phagosomal membrane damage (22). The shared features of cell wall remodelling, pyroptosis activation, and morphological transitions contribute to phagosomal membrane stress and represent intriguing fungal strategies in a race between microbe and phagocyte.

In both pathogenic fungi, the outcome of this intracellular phase is a race between fungal growth and host control, with much of the battle being waged over the integrity of the phagosomal membrane. In both cases, morphological transitions are associated with undermining phagosomal control, a process that might involve physical damage to the membrane. The precedents established by the studies of C. albicans and C. neoformans suggest the need for similar explorations with other pathogenic fungi. The ability for (i) asymmetric morphological transformation in the yeast-hyphal transition in dimorphic fungi and (ii) symmetrical spherical enlargement in cryptococcal species potentially provides these distinct fungal pathogens with a common mechanism to disrupt phagosomes through physical stress. The work of Westman and colleagues is the first time that we have been privileged to see this race happening with unprecedented detail in real time, a finding that now opens the door to similar approaches for a variety of other pathogens. This work sets a standard that can be applied to other pathogenic fungi to dissect these critical temporal sequences and better understand the process of intracellular pathogenesis.
